# A rare case report of laryngopharyngeal polyp formation following anterior cervical discectomy and fusion (ACDF)

**DOI:** 10.1186/s12891-020-03608-4

**Published:** 2020-09-12

**Authors:** Xiucheng Li, Lei He, Wei He, Zuo Lv, Xuerong Chen

**Affiliations:** Department of Orthopaedics, Shaoxing People’s Hospital (Shaoxing Hospital, Zhejiang University School of Medicine)Shaoxing, No. 568, Zhongxing North Road, Shaoxing, Zhejiang, 312000 People’s Republic of China

**Keywords:** Laryngopharyngeal polyp, Anterior cervical discectomy and fusion (ACDF), Esophageal fistula, Internal fixation removal, Polypectomy

## Abstract

**Background:**

Anterior Cervical Discectomy and Fusion (ACDF) has been regarded as the “gold standard” treatment of cervical spondylosis. Though it has good outcomes, many complications still exist, such as loss of fixation, degeneration of adjacent segments, dysphagia and pharyngeal perforation. In view of current literature, this study is the first to report a case of laryngopharyngeal polyp following ACDF.

**Case presentation:**

A 63 year old male patient suffered from cervical spine hyperextension after trauma accompanied by numbness of the hands and decreased muscle strength in both upper limbs. Anterior cervical fusion surgery was performed in our hospital, after which the patient’s upper limb numbness disappeared and muscle strength returned to normal. In the fifth month after surgery, the patient developed a sore throat and dysphagia. Symptoms gradually worsened, and the patient was hospitalized four times, subsequently undergoing tracheotomy, internal fixation removal, and polypectomy. The patient’s pronunciation, breathing, and swallowing functions returned to normal, and the incision healed. After a one-year follow-up, the polyp did not recur.

**Conclusions:**

Laryngopharyngeal polyp formation following ACDF has yet to be reported in literature. By excluding esophageal fistula as soon as possible, removing internal fixation and polypectomy serves as the best treatment in relieving patient symptoms.

## Background

Anterior Cervical Discectomy and Fusion (ACDF) was introduced by Smith and Robinson [1] in the 1950s. ACDF has been regarded as the “gold standard” treatment of cervical spondylosis even after half a century of its introduction [2]. ACDF has advantages in less surgical trauma, direct and thorough decompression, and effective restoration of the cervical physiological curvature. It has been widely used for treatment of fractures [3], instability [4], tumors [5], and other cervical spine disorders.

Many long-term follow-up studies have demonstrated excellent results. A 25-year follow-up study reported 81.1% of patients were free of radicular pain and had no repeated procedures. According to Odom’s criteria, 86.1% of good to excellent functional recovery was achieved [6]. After decades of surgical development, the fixation and fusion procedure as well as the materials required for ACDF have been updated and iterated. The use of plates in ACDF has shown improved clinical outcomes, fusion rates, stability and prevention of graft dislodgement as well as the restoration of the cervical physiological curvature [7]. Despite the relative safety and efficacy of ACDF, many complications such as soft-tissue injury [8], loss of fixation [9], degeneration of adjacent segments [10], dysphagia [11] and pharyngeal perforation [12] have been reported.

In this case report, a rare case of laryngopharyngeal polyp occurring following ACDF and presenting with progressive ventilation and dysphagia disorder is described. To the best of our knowledge, this is the first study reporting on the clinical presentation, diagnosis and treatment of laryngopharyngeal polyp after Anterior Cervical Discectomy and Fusion.

## Case presentation

**Fig. 1:**
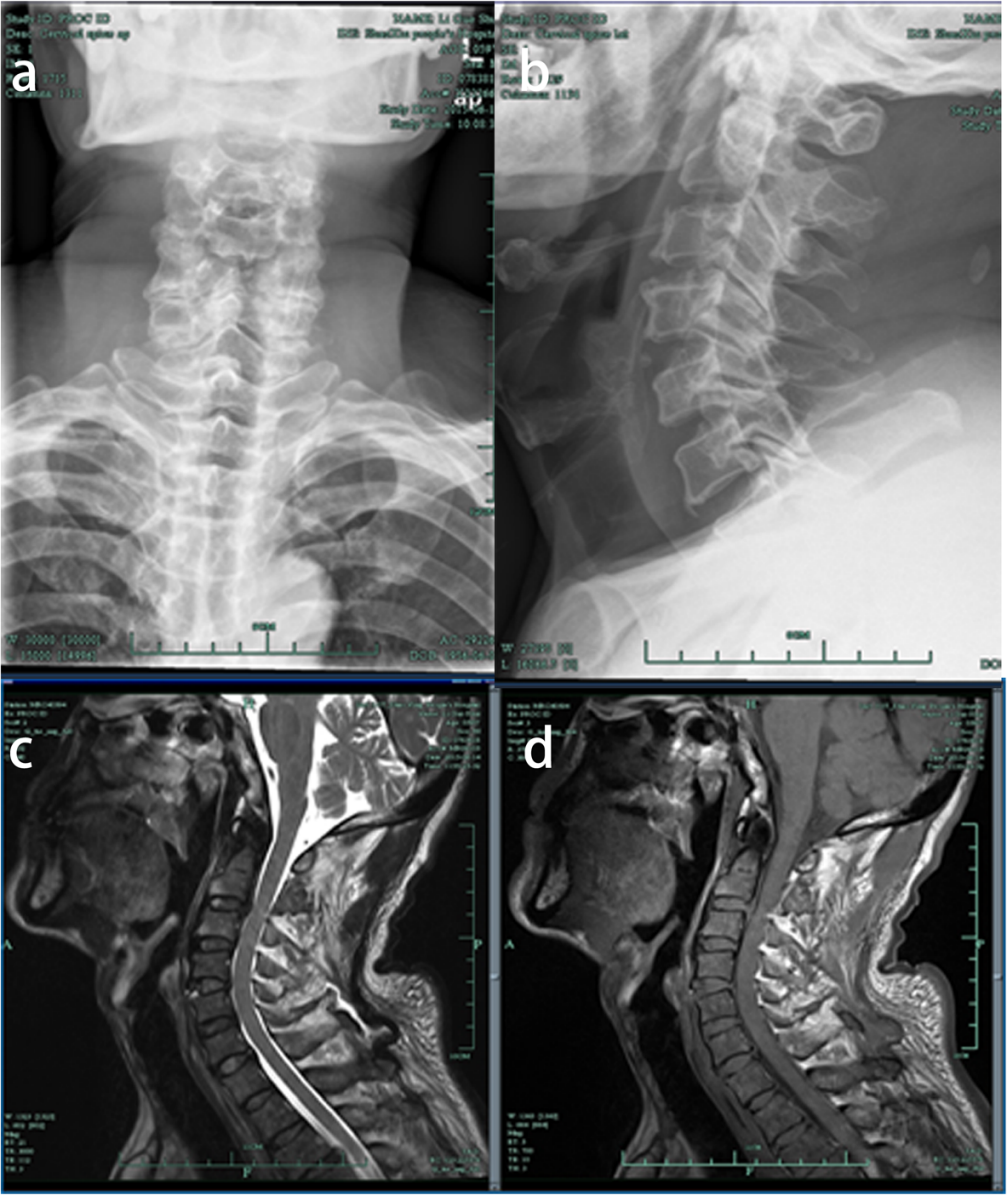
Imaging after first admission. **a** Anterior–posterior radiograph of the cervical spine demonstrating multilevel degenerative disc disease and facet arthropathy**. b** Lateral radiograph of the cervical spine showing an enlarged intervertebral space between C4–5 and degenerative changes of the spine. **c** T2-weighted sagittal image revealing anterior vertebral high signal and spinal cord signal changes at the C4/5 level. **d** There are no abnormalities in the throat

**Fig. 2:**
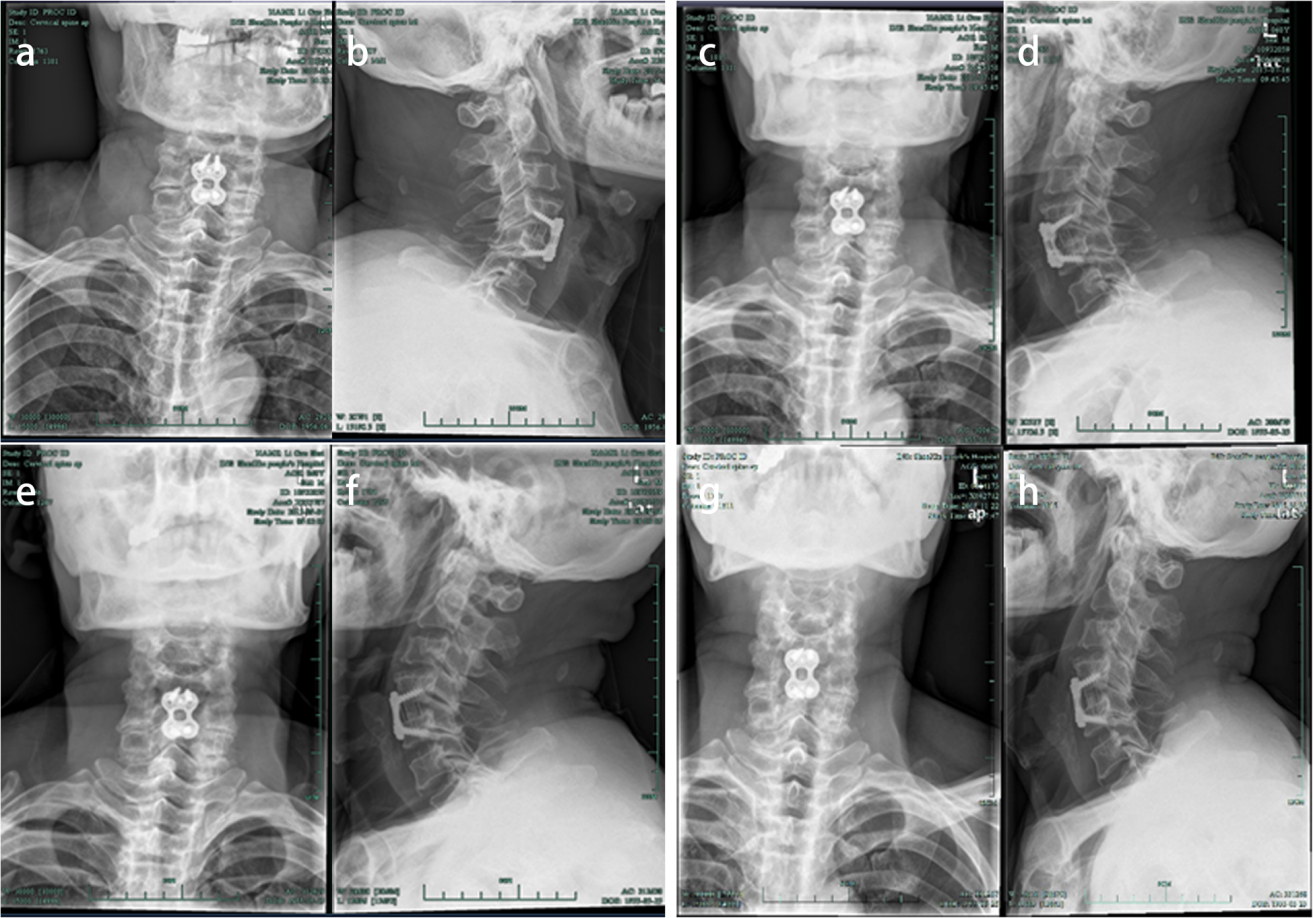
Images of cervical spine radiograph after ACDF showed that the position and length of the plate and screws were satisfactory without looseness or displacement**. a**, **b** One day after operation. **c**, **d** One month. **e**, **f** Three months**. g**, **h** Five months

A 63-year-old male farmer was admitted to our hospital with a 5-day history of weakness and numbness in both his upper limbs. Five days prior to admission, he accidentally slipped from a height of 2 m when going uphill to cut firewood. After a short rest, he went home by himself. However, his symptoms gradually worsened, where he had weakness, numbness and discomfort in both upper limbs for which he sought acupuncture for both hands. After admission, his physical examination revealed that the muscle strength of both upper limbs was 3/5, the two-hand grip strength was 2/5, and the superficial sensation of both upper limbs were disordered. Hoffman’s sign was negative. After a week of hormonal treatment, the patient’s symptoms did not improve significantly. Cervical X-ray showed his anterior space slightly stretched at the C4/5 stage (Fig. 1a, b). Magnetic resonance imaging (MRI) revealed anterior vertebral high signal and spinal cord signal changes at the C4/5 level (Fig. 1c, d), indicating anterior longitudinal ligament injury combined with instability. The patient then underwent ACDF at the C4–5 stage, which was performed unremarkably. Postoperative X-ray of his cervical spine showed that the position and length of the plate and screws were satisfactory (Fig. 2a, b). The symptoms of weakness and numbness in both his upper limbs gradually recovered. The patient had no fever, hoarseness, throat discomfort and dysphagia following surgery. Outpatient review of his cervical X-ray at one month (Fig. 2c, d) and three months (Fig. 2e, f) illustrated that the position of the plate and vertebral fusion cage did not change.

**Fig. 3:**
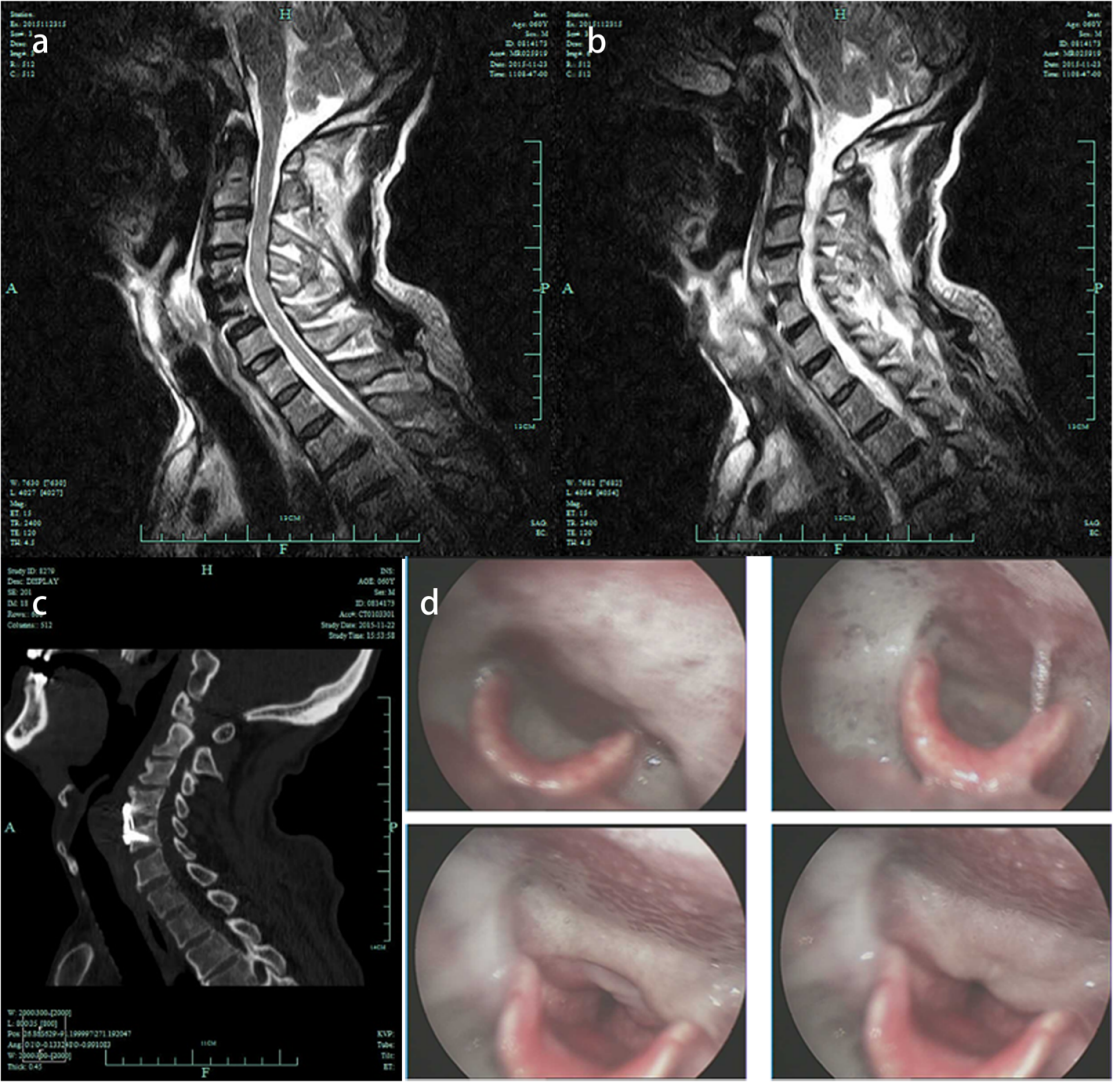
Imaging done five months after ACDF. **a, b** T2-weighted sagittal image demonstrated extensive high signal in throat tissue at the C4–5 level. **c** Computer tomography showed that the plate and cage were in their original positions without looseness or displacement. **d** Laryngoscope revealed edema and whiteness in the throat tissue without obstruction

Five months following surgery, the patient was admitted at our emergency department of Otolaryngology due to dysphagia. Both the patient himself and the medical records denied that the patient had medical history of laryngopharyngitis or other pharyngeal and laryngeal diseases before the operation. Cervical X-ray (Fig. 2g, h) and computer tomography (CT) (Fig. 3c) illustrated that his plate and cage were in the original position without looseness or displacement. Laryngoscopy revealed edema and whiteness in the throat tissue without obstruction (Fig. 3d), and cervical MRI demonstrated extensive high signal in the throat tissue (Fig. 3a, b). The patient had a low-grade fever, and lab work showed an increased C-reactive protein (CRP) of 13.48 mg/L. After treatment with antibiotics and hormones, his symptoms improved and he was discharged.

**Fig. 4:**
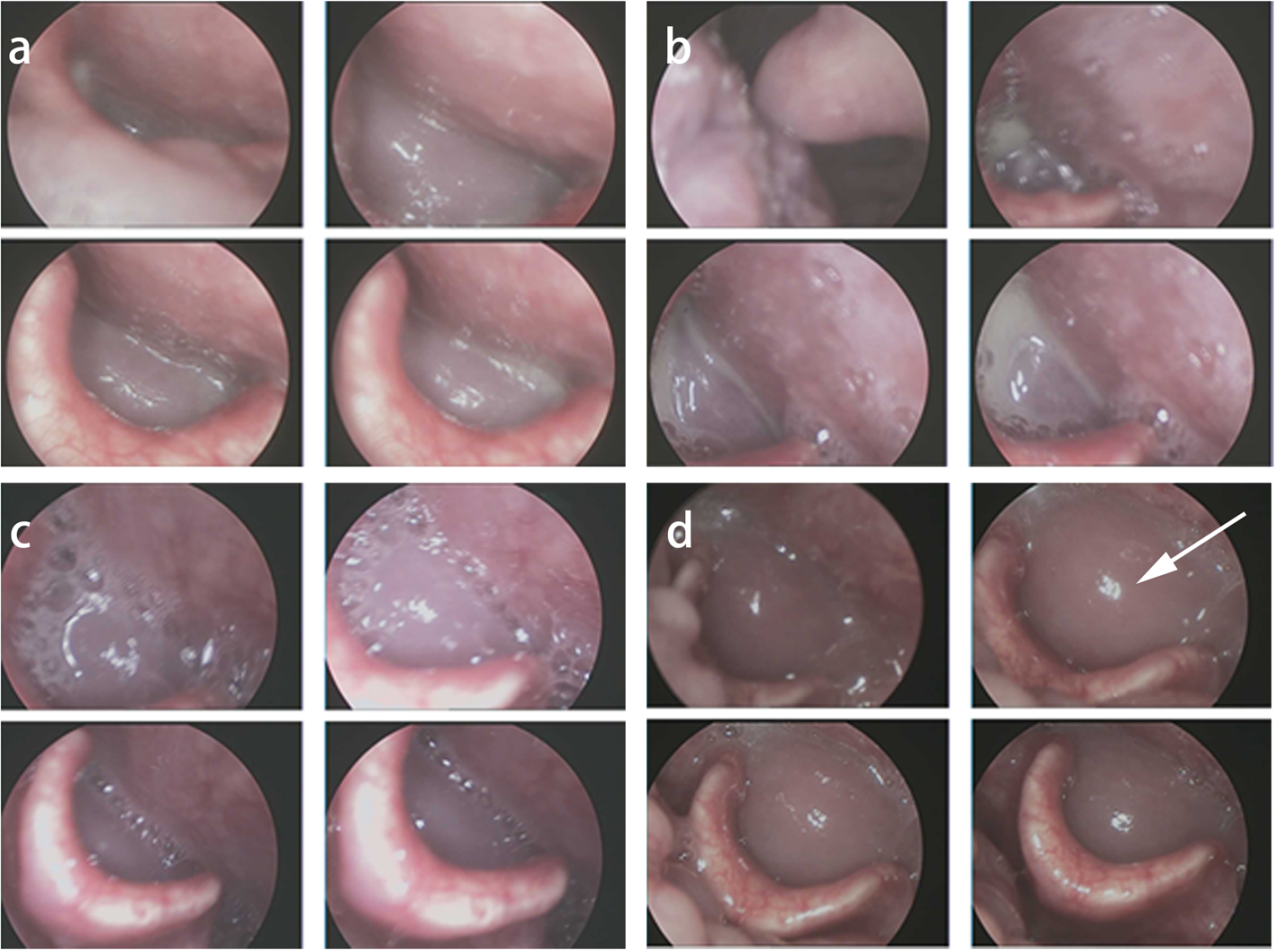
Images of the patient’s laryngoscope after ACDF. **a** 30 months. **b** 34 months. **c** 36 months. **d** 41 months. Larynx was completely blocked and epiglottis could not be closed (white arrow)

**Fig. 5:**
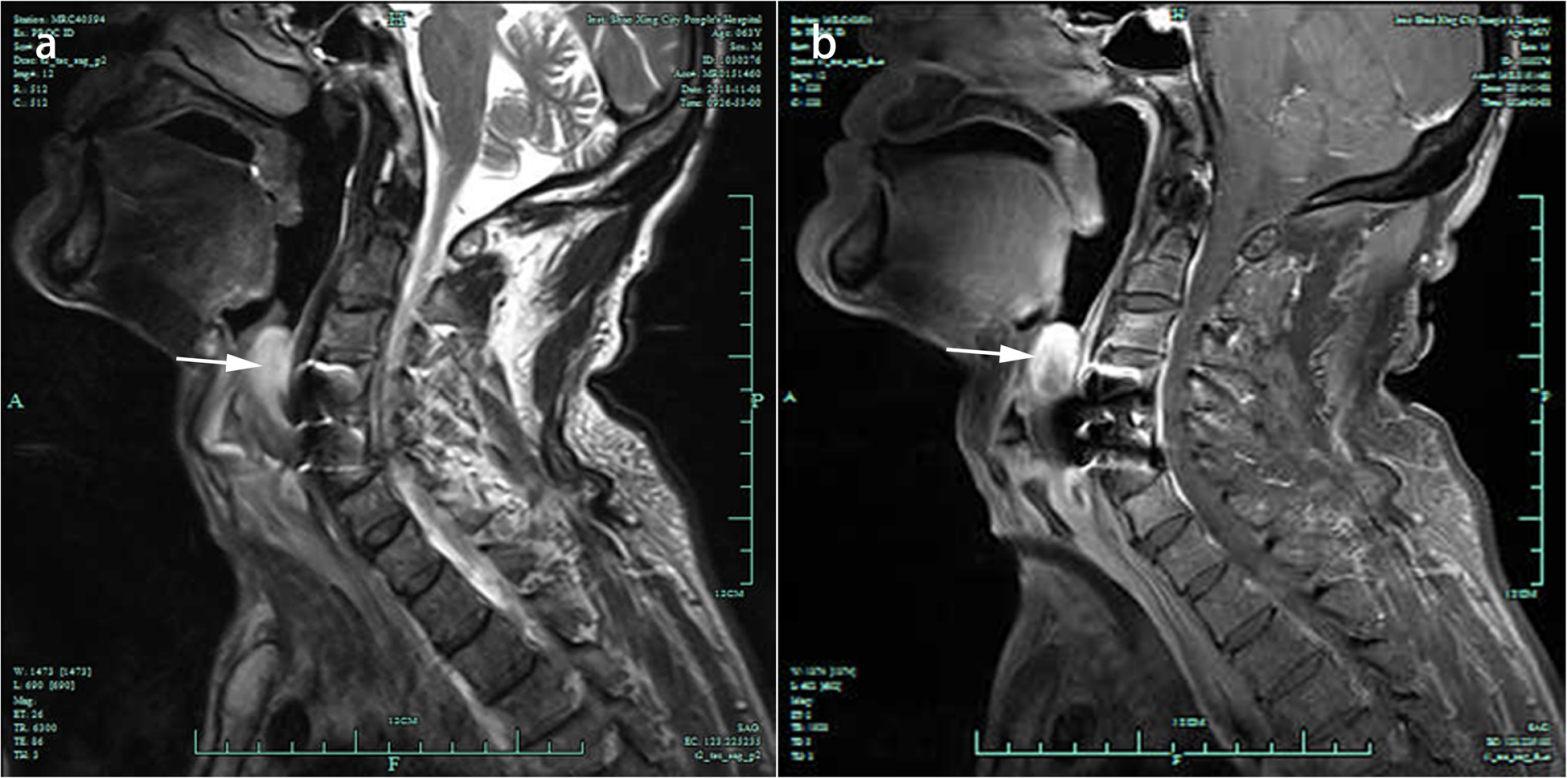
Images of Cervical MRI. **a, b** T2-weighted sagittal images revealed an oval mass of high-signal mass (white arrow) that completely blocked the throat

Our team later discovered that the patient did not recover after his initial symptoms of dysphagia. However, he did not return to the orthopedic clinic in our hospital. He returned two and a half years after the operation, where further laryngoscopy was done at 30 months (Fig. 4a), 34 months (Fig. 4b), 36 months (Fig. 4c), and 41 months (Fig. 4d) after ACDF. The patient had visited many other hospitals on suspicion of esophageal fistula for which he was recommended surgery, though he refused. The patient eventually was unable to swallow, drink liquids, and suffered from respiratory issues. These symptoms were very critical and endangered his life. As shown in Fig. 4d, his larynx was completely blocked and his epiglottis could not close. He had to undergo tracheotomy with an indwelling gastric tube. Cervical MRI also revealed a high-signal mass that completely blocked the throat (Fig. 5a, b).

**Fig. 6:**
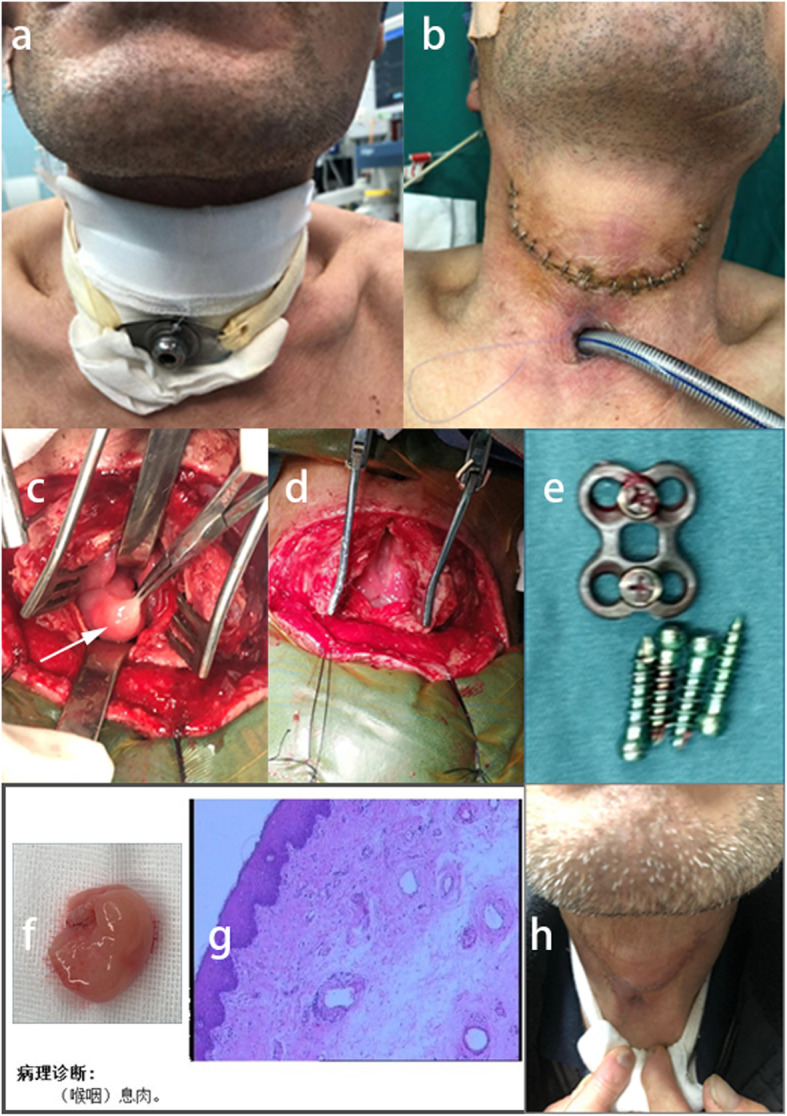
Images of Perioperative. **a, b** General appearance of the patient with tracheal intubation before the last operation. **c** A lump in the throat. **d** The posterior wall of the throat was unbroken. **e** Removed screws and plate. **f** Image of the specimen. **g** Pathological section of HE image. **h** General appearance of the patient after the last operation

After discussing with an otolaryngologist, a surgical plan was made in order to relieve the obstruction through the resection of the blockage in his throat while cleaning the larynx to explore the presence of an esophageal fistula, remove the internal fixation and prepare for direct primary closure. After a long period of low nutrition, the patient was very thin and was ventilated via tracheal intubation (Fig. 6a, b). A neck dissection was then performed by the otolaryngologist. When cutting the laryngeal knot, a smooth lump emerged and completely occupied the throat, blocking the entrance of the esophagus and trachea (Fig. 6c). Unexpectedly, when the lump was removed, no esophageal leakage was found. The posterior wall of the throat was unbroken, without sinuses or purulent fluid (Fig. 6d). Through the incision of the posterior wall of the throat, the internal fixation was removed. There were no signs of looseness or infection, and the surrounding tissue around the plate was notably edematous (Fig. 6e). The pathological diagnosis of the resected specimen was polyp formation (Fig. 6f, g). After a month’s rest, the incision healed (Fig. 6h), and the patient’s respiration and diet returned to normal. Furthermore, laryngoscopy showed no recurrence of polyps.

## Discussion and conclusions

This study documented a case of laryngopharynx polyps that occurred more than two years after ACDF. This is the first such report that aims to prevent misdiagnoses and delayed treatment for patients with similar presentations. Patients have suffered intolerable pain, which has caused serious consequences. In fact, ACDF has a history of more than 60 years, and many complications have been reported. The overall incidences of hoarseness and dysphagia after ACDF were reported to be 12.3 and 4.9%, respectively. A statistically significant increased risk of dysphagia has been reported after ACDF [13]. The incidence of pharyngoesophageal perforation varies between 0.25 and 1.49% [14]. Other complications of soft-tissue injury [8], nerve injury, loss of fixation [9], degeneration of adjacent segments [10], screw plate migration and pullout, and pharyngeal perforation [12] have been documented.

Irritation and injury of the esophagus following ACDF has been described since the advent of the procedure. Most reports involved the development of fistulas during the early postoperative period rather than delayed fistulas. A majority of patients with fistulas recovered in the early postoperative period without pharyngeal complaints. Moreover, zero profile devices reduced the stimulation of the esophagus as well as esophageal complications to a certain extent. A minority of patients, however, can develop into intractable and complicated cases. Esophageal fistula may be the first and most worrying complication for cervical surgeons. Esophageal fistula usually presents with symptoms of odynophagia, dysphagia, high fever, as well as salivary leakage from the incision site after eating [14]. Laboratory and radiological examinations such as MRI, esophageal radiography, and esophagoscopy are crucial in obtaining evidence for its diagnosis.

When the patient was finally hospitalized, a definite diagnosis was still unclear prior to operation. Before surgery, we fully prepared and discussed his case. It will be beneficial to discuss such cases with the otolaryngologist. For example, an uncommon incision was used by cutting the throat in this operation. Orthopedic surgeons are not good in making this incision. Hence, it is helpful to reveal the obstacle directly and reach the front of the throat and plate since in cases of chronic esophageal fistula, entering through the original surgical incision may be not the best choice as the adhesion around the esophagus will be severe and prevent adequate separation and exposure. It may be impossible to remove the internal fixation and perform an effective cleanup of infected tissue or repair the sinus. The main purpose of this operation was to detect and relieve the obstructive symptoms while removing the plate and preparing for muscle flap repair if needed. Fortunately, we found that the patient’s esophagus was free of infection and sinuses. Additionally, there were no signs of looseness or infection around the plate, though the surrounding tissue was edematous. The pathological result of the resection specimen was the formation of a laryngopharyngeal polyp. Benign neoplasms of the laryngopharynx are extremely rare [15]. Polyp is an abnormal tissue that resembles a mushroom but usually grows in the colon. Since they have abnormal cell growth, most polyps are benign. Polyps arise due to their location; sometimes no obvious incentive can determine the cause of polyps. Throat polyps are usually caused by injury from shouting loudly, damage from a breathing tube or inflammation. The cause of polyp formation in this case may be multifactorial as it developed slowly and gradually, causing symptoms of obstruction. First, the posterior wall of the esophagus may have been slightly damaged during surgery as it did not heal well and caused local inflammation. Second, as a foreign object, the plate continuously squeezed the posterior wall of the throat for an extended duration, causing ischemia and inflammation of the mucosa and muscles. Finally, the plate and screws may release metallic ions that stimulate the posterior wall of the throat, causing inflammation, which should not be excluded. In the early stages, the use of hormones could effectively alleviate symptoms and further confirm that inflammatory stimuli resulted in the production of polyps.

The key point in the present case was how to establish a clear diagnosis. When the polyp was removed, the patient quickly returned to normal. After three years, the patient suffered both physically and mentally, where he even risked death. This case report will serve as a guide for doctors to better comprehensively understand the postoperative complications of ACDF.

This case report documented a rare case of chronic laryngopharyngeal polyp formation following ACDF. It should pay more attention to the differential diagnosis of laryngopharyngeal polyp along with esophageal fistula and other throat tumors. Removal of polyps is an effective treatment, though its etiology is complicated. The authors of this study believe that its etiology is related to risk factors such as esophageal irritation during surgery, compression of the esophagus by the plate, and release of ions from internal fixation.

## Data Availability

All data generated or analyzed during this study are included in this published article.

## Abbreviations

ACDF: Anterior Cervical Discectomy and Fusion
CT: computer tomography
MRI: Magnetic resonance imaging
CRP: C-reactive protein
ENT: Ear, nose, and throat
